# Treatment efficacy and safety of regorafenib plus drug-eluting beads-transarterial chemoembolization versus regorafenib monotherapy in colorectal cancer liver metastasis patients who fail standard treatment regimens

**DOI:** 10.1007/s00432-021-03708-1

**Published:** 2021-07-24

**Authors:** Fei Cao, Jiaping Zheng, Jun Luo, Zhewei Zhang, Guoliang Shao

**Affiliations:** Department of Interventional Radiology, The Cancer Hospital of the University of Chinese Academy of Sciences (Zhejiang Cancer Hospital), Institute of Basic Medicine and Cancer (IBMC), Chinese Academy of Sciences, 1 Banshan Dong Lu, Gongshu District, Hangzhou, 310022 Zhejiang China

**Keywords:** Regorafenib plus DEB-TACE, CRLM, Treatment response, Prognosis, Adverse events

## Abstract

**Objective:**

This study aimed to evaluate the efficacy and safety of regorafenib plus drug-eluting beads-transarterial chemoembolization (DEB-TACE) versus regorafenib monotherapy in colorectal cancer liver metastases (CRLM) patients who failed standard treatment regimens.

**Methods:**

Totally, 76 eligible CRLM patients were analyzed, among which 42 patients received regorafenib monotherapy (as regorafenib group) and 34 patients received regorafenib plus DEB-TACE (as regorafenib plus DEB-TACE group).

**Results:**

Objective response rate (35.3% versus 7.1%, *P* = 0.002) and disease control rate (76.5% versus 47.6%, *P* = 0.011) were both increased in regorafenib plus DEB-TACE group compared with regorafenib group; meanwhile, negative conversion rate of carcinoembryonic antigen (66.7% versus 28.6%, *P* = 0.008) after treatment was elevated in regorafenib plus DEB-TACE group compared with regorafenib group. Notably, progression-free survival (PFS) (median value: 7.6 versus 4.1 months, *P* < 0.001) and overall survival (OS) (median value: 15.7 versus 9.2 months, *P* < 0.001) were both higher in regorafenib plus DEB-TACE group compared with regorafenib group. Furthermore, liver function indexes (alanine transaminase, aspartate aminotransferase, and cholinesterase levels) after treatment were all similar between the two groups (all *P* > 0.05). In addition, the occurrences of upper abdominal distending pain (*P* < 0.001), nausea and vomiting (*P* = 0.002) and fever (*P* = 0.002) were higher in regorafenib plus DEB-TACE group compared with regorafenib group, while the majority of these adverse events were mild and tolerable.

**Conclusions:**

Regorafenib plus DEB-TACE is superior to regorafenib monotherapy regarding treatment response, PFS and OS, while induces tolerable post-embolization syndrome in CRLM patients who fail standard treatment regimens.

**Supplementary Information:**

The online version contains supplementary material available at 10.1007/s00432-021-03708-1.

## Introduction

Colorectal cancer (CRC) is the third most common cancer that annually causes more than 14 million new CRC cases and over 8 million deaths worldwide (Ferlay 2015). Meanwhile, it is suggested that about 30–50% of CRC patients have developed colorectal cancer liver metastases (CRLM) at diagnosis, which is one of the main causes of death in CRC patients (van der Geest et al. 2015). Currently, the resection of CRLM is the only potentially curative treatment strategy for CRLM patients, which could provide a median survival time of 28–46 months (Zarour 2017). For the unresectable CRLM patients, the combination of fluoropyrimidine-based chemotherapy and vascular endothelial growth factor inhibitors is recommended as the first- and second-line treatment, which has improved their prognosis to some extent; while there are still a proportion of unresectable CRLM patients fail standard treatment regimens (Kow 2019; Van Cutsem et al. 2010).

Regorafenib is a multi-kinase inhibitor, which could suppress the activity of kinases that participate in angiogenesis, tumorigenesis and formation of the tumor microenvironment (Ettrich and Seufferlein 2018). Currently, regorafenib is recommended as the third-line or above therapy for unresectable CRLM patients (Van Cutsem et al. 2010). However, previous clinical trials show that compared with placebo, regorafenib could only limitedly prolong the OS of CRLM patients who fail standard treatment regimens (Grothey 2013; Juan et al. 2017; Li 2015). Therefore, further pursuing treatment strategies with better efficacy might improve the outcome of CRLM patients who fail standard treatment regimens.

Transarterial embolization (TACE) is frequently applied to treat primary and metastatic liver cancer, which combines the intratumoral cytotoxic effect of antitumor agents and ischemia effect of embolization agents to achieve considerable therapeutic effect (Raoul et al. 2019). As a new generation of TACE technology, drug-eluting beads (DEB)-TACE uses microspheres as both drug carriers and embolization agents to realize more stable and persistent drug release, thus achieving a better therapeutic effect compared with conventional TACE (Melchiorre 2018). Previous studies suggest that DEB-TACE presents a good treatment efficacy as well as mild and tolerable adverse events (mainly abdominal pain and fever) in CRLM patients (Huppert et al. 2014; Martin 2009; Ngo 2019). However, the embolization by DEB-TACE could induce hypoxia effect in CRLM, which might upregulate angiogenesis, thus further facilitate tumor progression or recurrence (Giordano 2014; Sergio 2008). Considering that regorafenib is a multi-kinase inhibitor that could suppress angiogenesis and regulate tumor microenvironment (mentioned before), we hypothesized that regorafenib plus DEB-TACE might be a good choice for CRLM patients who fail standard treatment regimens; however, no previous study had been conducted to explore that.

Therefore, the aim of this study was to compare the treatment response, survival benefit and adverse events of regorafenib plus DEB-TACE versus (vs.) regorafenib monotherapy in CRLM patients who fail standard treatment regimens.

## Methods

### Patients

Between October 2016 and January 2019, 112 CRLM patients with failure of standard chemotherapy regimens treatment who were treated in Zhejiang Cancer Hospital were recruited in this study. Patients who met all of the following criteria were eligible for inclusion: (1) the primary lesion was confirmed as colorectal cancer by histology or cytology examination; (2) the intrahepatic lesion was proved to be a metastasis from colorectal cancer by histology, cytology, or imaging examination; (3) no other metastasis was found except liver metastasis; (4) disease was progressed after previous first- and second-line standard treatment regimens; (5) there was at least one evaluable lesion (lesion diameter ≥ 1 cm) in the liver; (6) age ≥ 18 years; (7) no severe basic diseases such as heart failure, renal failure, respiratory failure, and severe coagulation dysfunction; (8) Eastern Cooperative Oncology Group (ECOG) score ≤ 2 points; (9) life expectancy ≥ 3 months. Patients presenting with any of the following conditions were excluded from the study: (1) contraindications to study drugs; (2) combination with other therapies during the treatment period; (3) incomplete follow-up data, including imaging and hematology data; (4) lost to follow-up; (5) death attributable to non-tumor causes during the treatment period; (6) pregnant woman. This study was approved by the Medical Ethics Committee of the Zhejiang cancer hospital, China. All included patients provided signed informed consents prior to participation in the study.

### Baseline data collection

Demographics and disease features of patients were documented after baseline examinations, which included age, gender, location of the primary lesion, histopathological type, resection of the primary lesion, ECOG score, Child–Pugh stage, number of intrahepatic metastases, the largest size of intrahepatic metastatic tumor, rat sarcoma viral oncogene homolog (RAS) mutation status, carcinoembryonic antigen (CEA) level, and carbohydrate antigen 199 (CA199) level, liver biochemical indexes [alanine transaminase (ALT), aspartate aminotransferase (AST), and cholinesterase].

### Treatments

Totally, 64 patients who only received regorafenib treatment were categorized into the regorafenib group; 48 patients who received regorafenib plus DEB-TACE treatment were categorized into regorafenib plus DEB-TACE group. In the regorafenib group, regorafenib (Bayer Company, Leverkusen, North Rhine-Westphalia, Germany) was administered orally at an initial dose of 120 mg/day, continued for 3 weeks, then stopped for 1 week. Every 4–6 weeks was a treatment cycle. If disease progression or intolerant toxicity occurred, the regorafenib dosage was reduced to 80 mg/day, or the regorafenib treatment was discontinued. The intolerant toxicity included grade 3 or 4 hematologic toxicity, dermal toxicity, hypertension, and/or liver dysfunction, according to National Cancer Institute Common Terminology Criteria for Adverse Events (NCI-CTCAE) version 4.0. (2010). In the regorafenib plus DEB-TACE group, patients first received DEB-TACE therapy, followed by regorafenib treatment. The regorafenib treatment was started on days 3–5 after DEB-TACE therapy. The usage, dosage, and treatment cycle of regorafenib were as same as those in the regorafenib group. After the first DEB-TACE therapy, patients were assessed according to the reexamination of imaging and laboratory tests to determine whether repeated DEB-TACE treatment was necessary. Regorafenib was discontinued 3–5 days before the next DEB-TACE treatment.

### DEB-TACE procedures

The DEB-TACE procedures were as follows: (a) preoperative preparation: 120 mg irinotecan (Jiangsu Hengrui Pharmaceutical Co. LTD, Lianyungang, Jiangsu, China) was dissolved in 5 mL of injection water, or 5 mL of 5% dextran; a bottle of CalliSpheres^®^ microspheres (Callisyn Biomedical-Suzhou Inc., Suzhou, Jiangsu, China) with the diameter of 100–300 μm was used for loading the irinotecan solution for 30 min; after drug-loading, the CalliSpheres^®^ microspheres were mixed with non-ionic contrast media in a 1:1.1 ratio, followed by standing for 5 min. (b) operative procedures: the patient was supine on Digital Subtraction Angiography (DSA) operating table, then bilateral groin area skin was routinely disinfected, and the sterile towel was placed; the right femoral artery was punctured with Seldinger technique under 3 mL 1% lidocaine local anesthesia; a 5F arterial sheath was inserted, then a 4F RH catheter was inserted into the abdominal trunk to perform angiography and identify the blood-supply artery for tumor in the liver; a 3F catheter was entered into blood-supply artery for tumor using superselective technique; the prepared CalliSpheres^®^ microspheres were infused into the blood-supply artery of tumor for embolization at a speed of 1 mL/min; when the blood flow of the blood-supply artery of tumor was obviously slowed or stagnated, the embolization was temporarily stopped; after 5 min, DSA was performed again to determine whether the tumor was completely embolized or not; if there were still some incompletely embolized vessels, supplementary embolization was performed; if a bottle of drug-loaded microsphere was insufficient to achieve complete embolization due to abundant blood supply for tumor, the blank microsphere was added for embolization according to the patient’s intraoperative condition. (c) postoperative treatment: the patient was sent back to the ward after completion of embolization; the patient was instructed to lie on his back for 24 h, and the puncture site was bandaged and braked for 6 h; the patient was given routine symptomatic treatments such as liver protection, stomach protection, anti-vomiting, and analgesia.

### Efficacy and safety assessment

Treatment response assessment for liver metastatic lesions was performed by computed tomography (CT) and magnetic resonance imaging (MRI) after 2-cycle treatment (2–3 months after initiation of treatment). Treatment response was categorized according to Modified Response Evaluation Criteria in Solid Tumors criteria (Lencioni and Llovet 2010), which included complete response (CR), partial response (PR), stable disease (SD), and progression disease (PD). The objective response rate (ORR) was defined as CR + PR and the disease-control rate (DCR) was defined as CR + PR + SD. Tumor markers including CEA and CA199 were also assessed after 2-cycle treatment, and the negative conversion rate was calculated, which was defined as the percentage of patients with tumor markers changing from positive (CEA > 5.0 ng/mL, CA199 > 37.0 U/mL) to negative (CEA ≤ 5.0 ng/mL, CA199 ≤ 37.0 U/mL). Patients’ performance status after 2-cycle treatment was evaluated by the ECOG score (Azam et al. 2019). Patients were followed up monthly by image examinations to monitor disease status and evaluate survival. Progression-free survival (PFS) was defined as the duration from treatment to first documented disease progression, or death. Overall survival (OS) was defined as the duration from treatment to death. In addition, for safety assessment, patients’ liver function biochemical indexes including ALT, AST and cholinesterase were determined after treatment, and the adverse events that occurred during this study were documented and graded according to NCI-CTCAE version 4.0. (2010).

### Statistical analysis

**Fig. 1:**
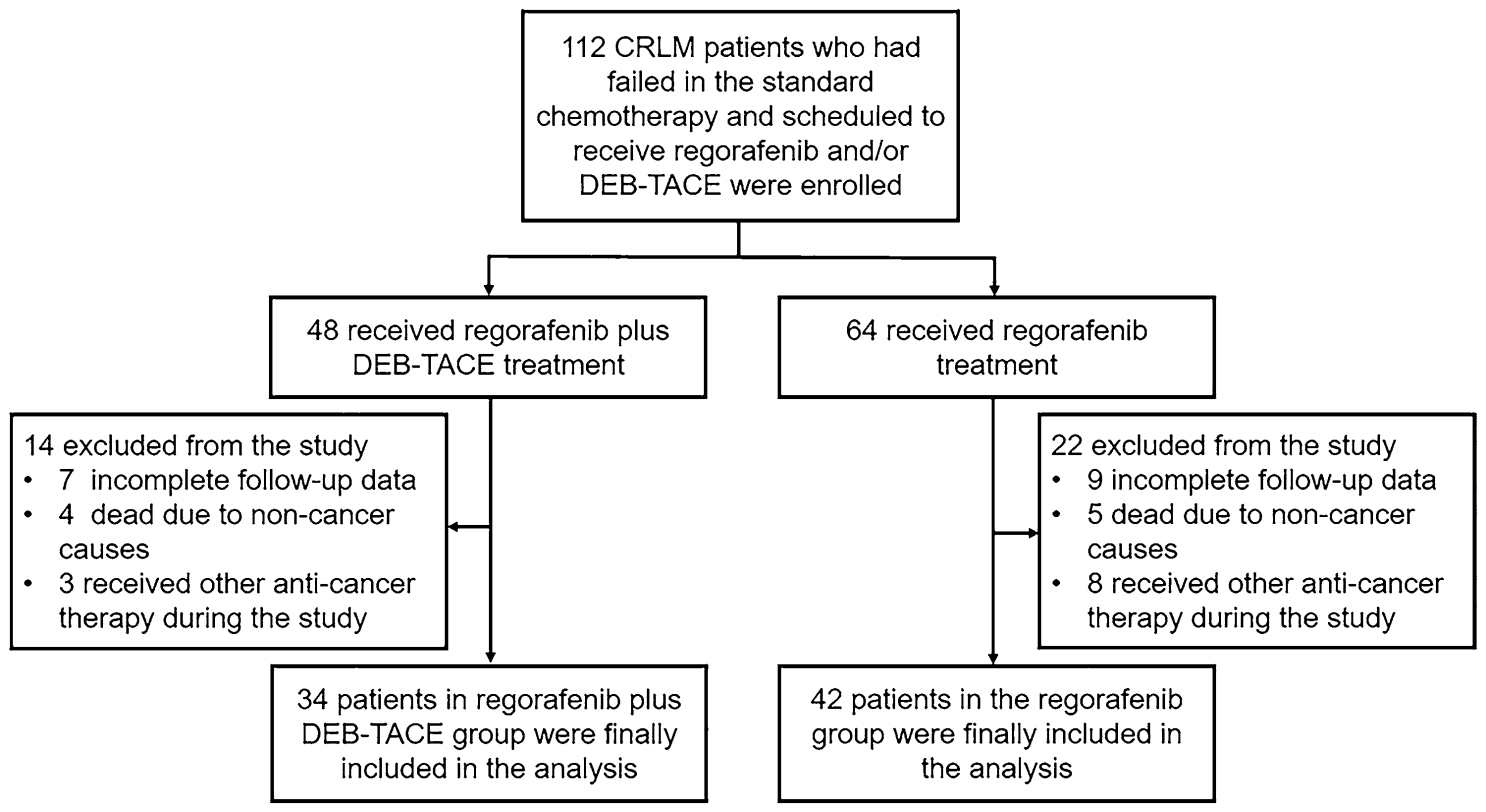
Study flow. *CRLM* colorectal cancer liver metastases, *DEB-TACE* drug-eluting beads-transarterial embolization

Finally, a total of 76 patients including 34 patients in the regorafenib plus DEB-TACE group and 42 patients in the regorafenib group were included in the analysis, and 36 patients were excluded from this study according to the exclusion criteria (Fig. 1). All statistical analyses were performed using SPSS software version 22.0 (IBM, Chicago, IL, USA). Quantitative data were displayed as mean with standard deviation (SD), and qualitative data were expressed as number with percentage [No. (%)]. Quantitative data and qualitative data were compared using *t* test and Chi-square test (including Fisher exact test), respectively. PFS and OS were displayed using Kaplan–Meier curves, and the difference in OS and PFS between the two groups was determined by the Log-rank test. *P* value < 0.05 was considered as statistically significant.

## Results

### Patients’ characteristics at baseline

In the regorafenib plus DEB-TACE group, the mean age of patients was 58.7 ± 7.3 years; there were 21 (61.8%) males as well as 13 (38.2%) females. Meanwhile, in the regorafenib group, the mean age of patients was 59.9 ± 7.2 years; there were 25 (59.5%) males as well as 17 (40.5%) females. Comparison analyses showed that liver function indexes including ALT, AST, and cholinesterase were all increased in regorafenib group compared with regorafenib plus DEB-TACE group (all *P* < 0.05), however, these indexes of the two groups were all within normal range. In addition, the Child–Pugh stage was similar between the two groups, implying that liver function were generally similar between the two groups. Besides, no difference was found in any other characteristics between the two groups (all *P* > 0.05) (Table 1).

**Table 1:** Comparison of clinical characteristics

Items	Regorafenib plus DEB-TACE (*N* = 34)	Regorafenib (*N* = 42)	*t*/*χ*^2^	*P* value
Age (years), mean ± SD	58.7 ± 7.3	59.9 ± 7.2	− 0.746	0.922
Gender, no. (%)			0.039	0.842
Male	21 (61.8)	25 (59.5)		
Female	13 (38.2)	17 (40.5)		
Primary nodule location, no. (%)			0.032	0.879
Rectum	22 (64.7)	28 (66.7)		
Colon	12 (35.3)	14 (33.3)		
Pathological type, no. (%)			0.838	0.360
Adenocarcinoma	33 (97.1)	40 (95.2)		
Mucinous carcinoma	1 (2.9)	1 (2.4)		
Adenosquamous carcinoma	0 (0.0)	1 (2.4)		
Primary nodule resection, no. (%)			0.006	0.936
Yes	24 (70.6)	30 (71.4)		
No	10 (29.4)	12 (28.6)		
Number of metastatic nodules in liver, no. (%)			0.246	0.620
Unifocal	8 (23.5)	12 (28.6)		
Multifocal	26 (76.5)	30 (71.4)		
Largest metastatic tumor size in liver, no. (%)			0.790	0.374
> 3.0 cm	28 (82.4)	31 (73.8)		
≤ 3.0 cm	6 (17.6)	11 (26.2)		
ECOG score, no. (%)			0.068	0.967
0	9 (26.5)	12 (28.6)		
1	18 (52.9)	21 (50.0)		
2	7 (20.6)	9 (21.4)		
Child–Pugh stage, no. (%)			0.127	0.721
A	23 (67.6)	30 (71.4)		
B	11 (32.4)	12 (28.6)		
Liver function biochemical indexes				
ALT (U/L), mean ± SD	13.1 ± 3.4	17.3 ± 9.6	− 2.638	0.011
AST (U/L), mean ± SD	21.2 ± 9.7	27.1 ± 6.0	− 3.099	0.003
Cholinesterase (U/L), mean ± SD	5697.8 ± 763.3	6350.9 ± 884.9	− 3.399	0.001
Tumor markers				
CEA, no. (%)			0.197	0.657
Positive (> 5.0 ng/mL)	21 (61.8)	28 (66.7)		
Negative (≤ 5.0 ng/mL)	13 (38.2)	14 (33.3)		
CA19, No. (%)			0.043	0.836
Positive (> 37.0 U/mL)	17 (50.0)	20 (47.6)		
Negative (≤ 37.0 U/mL)	17 (50.0)	22 (52.4)		
RAS mutation status, no. (%)			0.000	0.990
Wild	21 (61.8)	26 (61.9)		
Mutated	13 (38.2)	16 (38.1)		
Number of previous systemic anti-cancer therapies (on or after diagnosis of metastatic disease)			0.028	0.866
1–2	10 (29.4)	13 (31.0)		
3	8 (23.5)	10 (23.8)		
≥ 4	16 (47.1)	19 (45.2)		
Patients stopping previous treatment because of progression				
Oxaliplatin	19 (55.9)	26 (61.9)	0.282	0.595
Irinotecan	29 (85.3)	37 (88.1)	0.129	0.719
Fluoropyrimidine	28 (82.4)	35 (83.3)	0.013	0.910
Bevacizumab	24 (70.6)	31 (73.8)	0.098	0.755
Panitumumab or cetuximab, or both	10 (29.4)	12 (28.6)	0.006	0.936

*DEB-TACE* drug-eluting bead transarterial chemoembolization, *SD* standard deviation, *ECOG* Eastern Co-operative Oncology Group, *ALT* alanine transaminase, *AST* aspartate aminotransferase, *CEA* carcinoembryonic antigen, *CA199* carbohydrate antigen 199, *RAS* rat sarcoma viral oncogene homolog

Comparison was determined by Student’s *t* test or chi-squared test

### Treatment response

Patients’ treatment response after treatment was better in the regorafenib plus DEB-TACE group compared with the regorafenib group (*P* < 0.001). Specifically, in the regorafenib plus DEB-TACE group, 2 (5.9%) patients achieved CR, 10 (29.4%) patients achieved PR, 14 (41.2%) patients had SD and 8 (23.5%) patients had PD; meanwhile, in the regorafenib group, no patient achieved CR, 3 (7.1%) patients achieved PR, 17 (40.5%) patients had SD and 22 (52.4%) patients had PD. Besides, ORR (12 (35.3%) vs. 3 (7.1%), *P* = 0.002) and DCR (26 (76.5%) vs. 20 (47.6%), *P* = 0.011) were both increased in the regorafenib plus DEB-TACE group compared with regorafenib group (Table 2).

**Table 2:** Comparison of treatment response after treatment

Items	Regorafenib plus DEB-TACE (*N* = 34)	Regorafenib (*N* = 42)	*W*/*χ*^2^	*P* value
Total treatment response			1019.000	0.001
CR, no. (%)	2 (5.9)	0 (0.0)		
PR, no. (%)	10 (29.4)	3 (7.1)		
SD, no. (%)	14 (41.2)	17 (40.5)		
PD, no. (%)	8 (23.5)	22 (52.4)		
ORR, no. (%)	12 (35.3)	3 (7.1)	9.400	0.002
DCR, no. (%)	26 (76.5)	20 (47.6)	6.546	0.011

*DEB-TACE* drug-eluting bead transarterial chemoembolization, *CR* complete response, *PR* partial response, *SD* stable disease, *PD* progressive disease, *ORR* objective response rate, *DCR* disease control rate

Comparison was determined by Wilcoxon rank sum test or chi-squared test

### Negative conversion rate of tumor markers and ECOG score

Beside treatment response, the negative conversion rate of tumor markers and ECOG score were also used to compare the short-term treatment efficacy between the two groups. The negative conversion rate of CEA was higher in the regorafenib plus DEB-TACE group [14 out of 21 (66.7%) patients] compared with the regorafenib group [8 out of 28 (28.6%) patients] (*P* = 0.008). However, the negative conversion rate of CA199 was similar between the regorafenib plus DEB-TACE group [4 out of 17 (23.5%) patients] and the regorafenib group [5 out of 20 (25.0%) patients] (*P* = 0.495) (Table 3).

**Table 3:** Comparison of negative conversion rate of tumor markers after treatment

Item no. (%)	Regorafenib plus DEB-TACE	Regorafenib	*χ*^2^	*P* value
Negative conversion rate of CEA	14/21 (66.7)	8/28 (28.6)	7.039	0.008
Negative conversion rate of CA199	4/17 (23.5)	5/20 (25.0)	0.011	0.495

*DEB-TACE* drug-eluting bead transarterial chemoembolization, *CEA* carcinoembryonic antigen, *CA199* carbohydrate antigen 199

Comparison was determined by chi-squared test

Meanwhile, in the regorafenib plus DEB-TACE group, 11 (32.4%) patients had ECOG score 0, 17 (50.0%) patients had ECOG score 1 and 6 (17.6%) patients had ECOG score 2 after treatment; in the regorafenib group, 10 (23.8%) patients had ECOG score 0, 22 (52.4%) patients had ECOG score 1 and 10 (23.8%) patients had ECOG score 2 after treatment. The comparison analysis revealed that ECOG score after treatment was similar between the two groups (*P* = 0.652) (Table 4).

**Table 4:** Comparison of ECOG score after treatment

Items	Regorafenib plus DEB-TACE (*N* = 34)	Regorafenib (*N* = 42)	*χ*^2^	*P* value
ECOG score, no. (%)			0.865	0.652
0	11 (32.4)	10 (23.8)		
1	17 (50.0)	22 (52.4)		
2	6 (17.6)	10 (23.8)		

*DEB-TACE* drug-eluting bead transarterial chemoembolization, *ECOG* Eastern Co-operative Oncology Group

Comparison was determined by chi-squared test

### PFS and OS

**Fig. 2:**
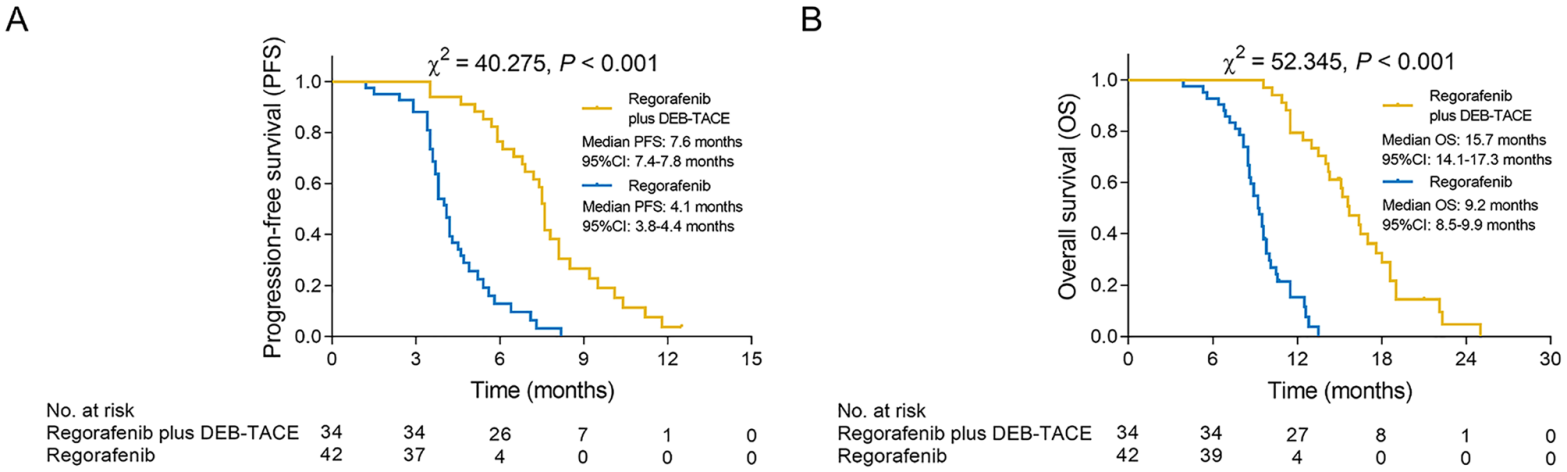
PFS and OS in regorafenib plus DEB-TACE group and regorafenib group. **A** Comparison of PFS between regorafenib plus DEB-TACE group and regorafenib group; **B** comparison of OS between regorafenib plus DEB-TACE group and regorafenib group. *PFS* progression-free survival, *OS* overall survival, *DEB-TACE* drug-eluting beads-transarterial embolization, *CI* confidence interval

PFS was elevated in the regorafenib plus DEB-TACE group (median value 7.6 months, 95% CI 7.4–7.8 months) compared with the regorafenib group (median value 4.1 months, 95% CI 3.8–4.4 months) (*P* < 0.001) (Fig. 2A). Meanwhile, OS was also increased in the regorafenib plus DEB-TACE group (median value 15.7 months, 95% CI 14.1–17.3 months) compared with the regorafenib group (median value 9.2 months, 95% CI 8.5–9.9 months) (*P* < 0.001) (Fig. 2B).

Furthermore, all patients were divided into subgroups according to their CRLM feature. In subgroup analyses, PFS and OS were all increased in regorafenib plus DEB-TACE group compared with the regorafenib group in single liver metastasis, multiple liver metastasis, tumor size < 3 cm, tumor size 3–5 cm, and tumor size > 5 cm subgroups (all *P* < 0.05) (Supplementary Table 1).

### Liver function indexes

In both regorafenib plus DEB-TACE group and regorafenib group, all patients had normal ALT and AST levels after treatment. Meanwhile, in the regorafenib plus DEB-TACE group, 5 (14.7%) patients had abnormal cholinesterase level while 29 (85.3%) patients had normal cholinesterase level; in the regorafenib group, 1 (2.4%) patient had abnormal cholinesterase level while 41 (97.6%) patients had normal cholinesterase level after treatment. Further comparison analyses did not find any difference in cholinesterase level after treatment between the two groups (*P* = 0.084) (Table 5).

**Table 5:** Comparison of liver function biochemical indexes after treatment

Items	Regorafenib plus DEB-TACE (*N* = 34)	Regorafenib (*N* = 42)	*χ*^2^	*P* value
ALT, no. (%)			–	–
Abnormal (> 40 U/L)	0 (0.0)	0 (0.0)		
Normal (≤ 40 U/L)	34 (100.0)	42 (100.0)		
AST, no. (%)			–	–
Abnormal (> 40 U/L)	0 (0.0)	0 (0.0)		
Normal (≤ 40 U/L)	34 (100.0)	42 (100.0)		
Cholinesterase, no. (%)			–	0.084
Abnormal (> 32,000 U/L or < 4300 U/L)	5 (14.7)	1 (2.4)		
Normal (4300–32,000 U/L)	29 (85.3)	41 (97.6)		

*DEB-TACE* drug-eluting bead transarterial chemoembolization, *ALT* alanine transaminase, *AST* aspartate aminotransferase

Comparison was determined by Fisher’s exact test

### Adverse events

Upper abdominal distending pain [21 (61.8%) vs. 0 (0.0%), *P* < 0.001], nausea, and vomiting [15 (44.1%) vs. (5 (11.9%), *P* = 0.002] and fever [7 (20.6%) vs. 0 (0.0%), *P* = 0.002] were more commonly occurred in the regorafenib plus DEB-TACE group compared with the regorafenib group; while no difference was found in the occurrence of other adverse events between the two groups (all *P* > 0.05). Meanwhile, no difference was found in the occurrence of grade ≥ 3 adverse events between the two groups either (all *P* > 0.05). Generally, the severity of adverse events was relatively mild, and only a few grade ≥ 3 adverse events occurred in both two groups (Table 6).

**Table 6:** Comparison of adverse events

Items, no. (%)	Regorafenib plus DEB-TACE (*N* = 34)		Regorafenib (*N* = 42)		*χ*^2^*	*P* value*	*χ*^2#^	*P* value^**#**^
	Total	Grade ≥ 3	Total	Grade ≥ 3				
Upper abdominal distending pain	21 (61.8)	0 (0.0)	0 (0.0)	0 (0.0)	35.846	< 0.001	–	–
Hand-foot-skin reaction	16 (47.1)	6 (17.6)	22 (52.4)	6 (14.3)	0.213	0.645	0.160	0.689
Nausea and vomiting	15 (44.1)	2 (5.9)	5 (11.9)	2 (4.8)	10.055	0.002	0.047	0.828
Fatigue	15 (44.1)	4 (11.8)	19 (45.2)	5 (11.9)	0.010	0.922	–	0.635
Hypertension	10 (29.4)	1 (2.9)	14 (33.3)	3 (7.1)	0.134	0.715	0.665	0.415
Diarrhea	9 (26.5)	3 (8.8)	11 (26.2)	4 (9.5)	0.001	0.978	0.011	0.916
Anorexia	7 (20.6)	0 (0.0)	9 (21.4)	1 (2.4)	0.008	0.929	–	0.553
Fever	7 (20.6)	0 (0.0)	0 (0.0)	0 (0.0)	9.524	0.002	–	–
Oral mucositis	6 (17.6)	1 (2.9)	7 (16.7)	3 (7.1)	0.013	0.910	0.665	0.415
Lose weight	4 (11.8)	1 (2.9)	6 (14.3)	0 (0.0)	0.105	0.746	–	0.447
Thrombocytopenia	4 (11.8)	0 (0.0)	4 (9.5)	1 (2.4)	0.100	0.752	–	0.553
Emesis	3 (8.8)	0 (0.0)	5 (11.9)	0 (0.0)	0.189	0.663	–	–
Proteinuria	3 (8.8)	0 (0.0)	5 (11.9)	0 (0.0)	0.189	0.663	–	–
Headache	2 (5.9)	0 (0.0)	4 (9.5)	0 (0.0)	0.343	0.558	–	–
Stomachache	2 (5.9)	0 (0.0)	3 (7.1)	0 (0.0)	0.049	0.826	–	–
Hypophosphatemia	2 (5.9)	0 (0.0)	3 (7.1)	0 (0.0)	0.049	0.826	–	–

Comparison was determined by chi-squared test or Fisher’s exact test

*DEB-TACE* drug-eluting bead transarterial chemoembolization

*Comparison of total adverse events between regorafenib plus DEB-TACE group and regorafenib group. ^#^Comparison of grade ≥ 3 adverse events between regorafenib plus DEB-TACE group and regorafenib group

### Representative case presentation

Besides, we here presented a representative CRLM case who achieved CR after regorafenib plus DEB-TACE treatment. The patient was a 40-year-old female who was diagnosed as multifocal CRLM. The computed tomography (CT) scan showed that there were two intrahepatic metastatic lesions and the diameter of the larger lesion was about 2.7 cm (Supplementary Fig. 1A, B). The patient then received regorafenib plus DEB-TACE treatment. Three months after treatment, repeated CT scan revealed that both of the two intrahepatic lesions were smaller than the initiation of the treatment (Supplementary Fig. 1C, D). Six months after treatment, the repeated CT scan illustrated that both of the intrahepatic lesions were almost completely necrotic, and the patient achieved CR (Supplementary Fig. 1E, F).

## Discussion

DEB-TACE, which possesses improved treatment efficacy and less systematic toxicity compared with conventional TACE, is vastly performed in patients with primary liver tumors (Melchiorre et al. 2018). Meanwhile, it is also suggested that DEB-TACE may be an effective treatment in patients with metastatic liver tumors, such as patients with neuroendocrine tumor liver metastases, melanoma liver metastases or CRLM (Carling 2015; Do Minh 2017; Huppert et al. 2014). Notably, one previous study shows that DEB-TACE treatment achieves a median PFS of 5 months and a median OS of 8 months in CRLM patients who fail standard treatment regimens (Huppert et al. 2014). Another multicenter, single-arm study reveals that CRLM patients who receive DEB-TACE treatment after failure of standard treatment regimens present a median DFS of 247 days and a median OS of 343 days (Martin et al. 2009). Moreover, a randomized, single-center trial suggests that DEB-TACE improves the OS (median value 16 vs. 13 months) of CRLM patients who fail first- and second-line treatment compared with conventional TACE (Vogl et al. 2020). Therefore, it could be deduced that DEB-TACE might be a promising strategy for CRLM patients who fail standard treatment regimens, while its potential in combining with regorafenib as the third-line or above treatment for the unresectable CRLM patients is still unclear.

In the present study, regorafenib plus DEB-TACE treatment achieved higher ORR and DCR compared with regorafenib monotherapy, meanwhile, regorafenib plus DEB-TACE treatment increased the negative conversion rate of tumor marker CEA compared with regorafenib monotherapy in CRLM patients who fail standard treatment regimens. These data could be explained by that the embolization effect of DEB-TACE induced hypoxia to increase the necrotic CRC cells that metastasized to the liver; additionally, the persistent locoregional cytotoxicity effect of the chemotherapeutic agents released by DEB-TACE also increased the necrotic CRC cells that metastasized to the liver (Melchiorre et al. 2018); besides, considering that regorafenib is a multi-kinase inhibitor that could suppress angiogenesis, regorafenib plus DEB-TACE not only took the advantage of DEB-TACE (persistent embolization and chemotherapy) but also ameliorated the embolization-induced upregulation of angiogenesis to exert good treatment efficacy; thus, compared with regorafenib monotherapy, regorafenib plus DEB-TACE treatment could achieve a higher response rate to promote tumor remission, which further resulted in a higher negative conversion rate of tumor marker CEA in CRLM patients who fail standard treatment regimens. Meanwhile, it was found that regorafenib plus DEB-TACE treatment did not vary ECOG score after treatment compared with regorafenib monotherapy in CRLM patients who fail standard treatment regimens, which could be explained by that although regorafenib plus DEB-TACE treatment achieved higher treatment response and negative conversion rate of CEA compared with regorafenib monotherapy, it might not change patients’ performance status in a short period of time. Moreover, we observed that regorafenib plus DEB-TACE treatment prolonged PFS and OS compared with regorafenib monotherapy in CRLM patients who fail standard treatment regimens. Possible explanations for our data might be that: the persistent locoregional chemotherapy and embolization by DEB-TACE could ameliorate the progression of CRLM (Martin et al. 2009; Vogl et al. 2020), thus regorafenib plus DEB-TACE elongated the PFS of CRLM patients who fail standard treatment regimens compared with regorafenib monotherapy; besides, regorafenib plus DEB-TACE achieved higher treatment response compared with regorafenib monotherapy, thus prolonging the OS of CRLM patients who fail standard treatment regimens.

The adverse events of regorafenib or DEB-TACE in CRLM patients have been reported by previous studies. For instance, as to the adverse events related to regorafenib treatment, the CORRECT trial suggests that in the unresectable CRLM patients, the most commonly occurred adverse events are fatigue, hand-food-skin reaction and diarrhea, and the most commonly occurred grade ≥ 3 adverse events are hand-food-skin reaction and fatigue (Grothey et al. 2013). Meanwhile, other studies suggest that the most commonly occurred adverse events related to DEB-TACE is abdominal pain, nausea, and vomiting, as well as fever, while grade ≥ 3 adverse events related to DEB-TACE rarely occur in patients with metastatic liver tumor (Fiorentini et al. 2008, 2009). Partly in line with previous studies (Fiorentini et al. 2008, 2009), we found that the most commonly occurred adverse events related to regorafenib were hand-food-skin reaction and fatigue, while those related to DEB-TACE was upper abdominal distending pain in CRLM patients who fail standard treatment regimens. Notably, regorafenib plus DEB-TACE treatment induced more upper abdominal distending pain, nausea, and vomiting, as well as fever compared with regorafenib monotherapy in CRLM patients who fail standard treatment regimens, among which upper abdominal distending pain and fever might be related to the necrosis and inflammation in tumor tissues induced by the hypoxia effect of embolization of DEB-TACE (Fiorentini et al. 2008; Fiorentini et al. 2009), and nausea and vomiting could be induced by irinotecan loaded by DEB-TACE, which is well-illustrated by previous studies (Affronti 2017; Suzuki et al. 2016). Besides, regorafenib plus DEB-TACE treatment did not induce additional grade ≥ 3 adverse events or abnormality in liver function compared with regorafenib treatment monotherapy, which implied that regorafenib plus DEB-TACE treatment was generally tolerable in CRLM patients who failed standard treatment regimens.

In the present study, patients who lost follow-up, patients who died due to non-cancerous reasons and patients who received other anti-cancer therapy during the study were excluded from final analyses. The reasons were listed as follows: (1) for patients who lost follow-up: most patients lost follow-up in the early phase of this study, thus they had no assessment data; (2) for patients who died due to non-cancerous reasons: they had no data on hepatic lesion progression, thus it was unable to assess PFS in these patients; (3) for patients who received other anti-cancer therapies during the study: it was meaningless to assess PFS in these patients, besides, we did not collect data of these patients for they received other therapies. Therefore, we excluded these patients from final analyses. Thus, we conducted analyses according to PP set but not ITT set.

There existed several limitations in this study. First of all, the sample size of this study was relatively small, which might cause low statistical power. Therefore, further studies with larger sample sizes should be conducted to verify the efficacy and safety of regorafenib plus DEB-TACE in CRLM patients who failed standard treatment regimens. Next, this study was a single-center study, which might cause regional bias, and further multicenter studies could be conducted to avoid regional bias. Moreover, since this was an observational study, a large number of patients lost follow-up or changed treatment strategy and some of them were excluded from analyses; besides, although no difference was found in the clinical characteristics between the two groups at baseline, the confounding factors still objectively existed since this was an observational study. Thus, further well-designed clinical trials were encouraged to verify the potential of DEB-TACE plus regorafenib as the third-line or above treatment for the unresectable CRLM patients.

To be conclusive, regorafenib plus DEB-TACE is superior to regorafenib monotherapy regarding treatment response, PFS and OS, while it induces mild post-embolization syndrome to some extent in treating CRLM patients who fail standard treatment regimens. This study suggests that regorafenib plus DEB-TACE might be an alternative option for CRLM patients who fail standard treatment regimens.

## Supplementary Information

Below is the link to the electronic supplementary material.

Supplementary file1 CT scan images focusing on CRLM lesions before and after regorafenib plus DEB-TACE treatment from a representative case. A: CRLM lesion 1 before treatment; B: CRLM lesion 2 before treatment; C: CRLM lesion 1 three months after treatment; D: CRLM lesion 2 (TIF 4117 KB)

Supplementary file2 (DOCX 19 KB)

## Data Availability

The datasets used and/or analyzed during the current study are available from the corresponding author on reasonable request.
