# Supplementary figures and images for: Treatment efficacy and safety of regorafenib plus drug-eluting beads-transarterial chemoembolization versus regorafenib monotherapy in colorectal cancer liver metastasis patients who fail standard treatment regimens
Source: J Cancer Res Clin Oncol. 2021 Jul 24;147(10):2993–3002. doi: 10.1007/s00432-021-03708-1 (PMC8397621; doi:10.1007/s00432-021-03708-1)

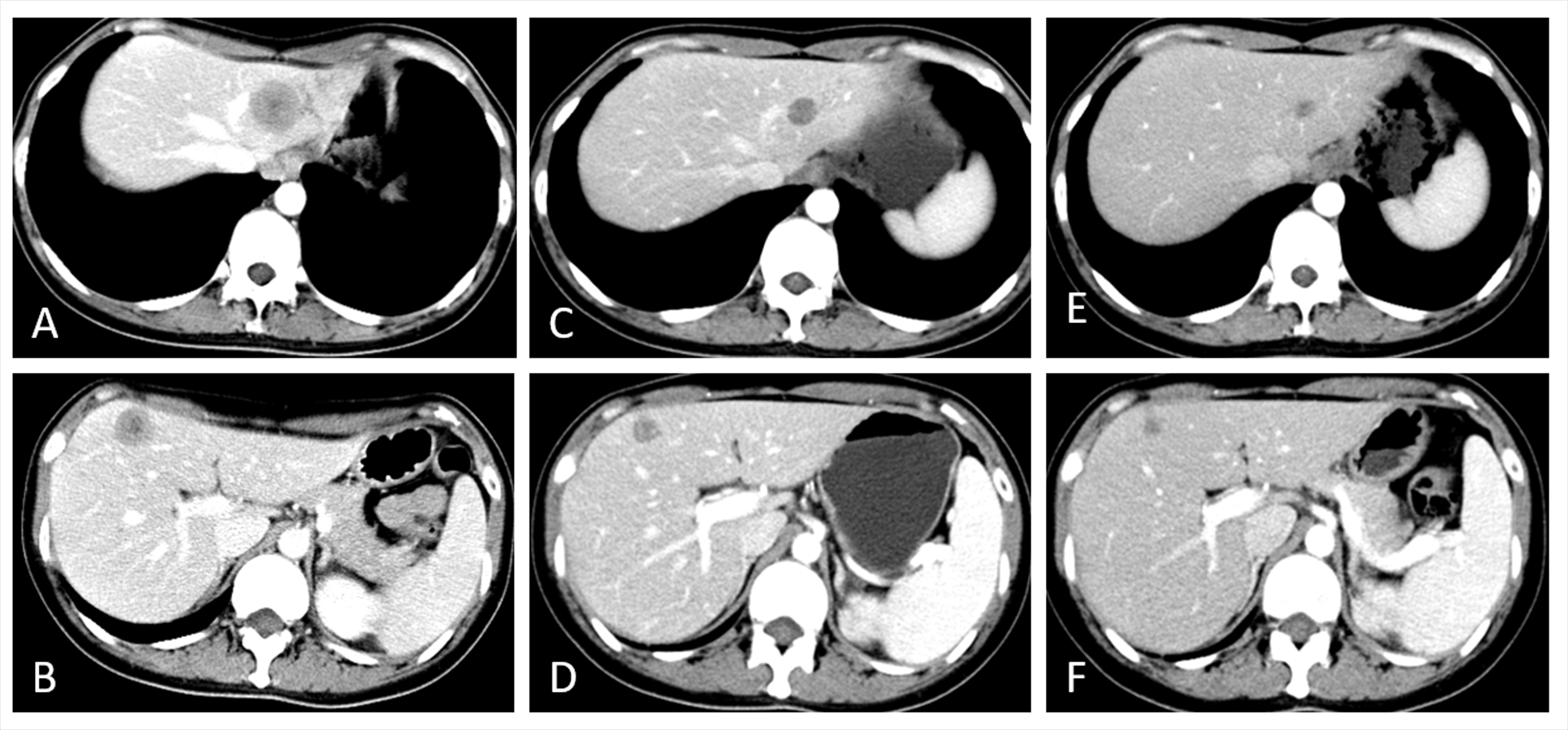

Supplement: Supplementary file 1 — Supplementary file1 CT scan images focusing on CRLM lesions before and after regorafenib plus DEB-TACE treatment from a representative case. A: CRLM lesion 1 before treatment; B: CRLM lesion 2 before treatment; C: CRLM lesion 1 three months after treatment; D: CRLM lesion 2 (TIF 4117 KB) [file 432_2021_3708_MOESM1_ESM.tif]
