# Supplementary material for: Treatment efficacy and safety of regorafenib plus drug-eluting beads-transarterial chemoembolization versus regorafenib monotherapy in colorectal cancer liver metastasis patients who fail standard treatment regimens
Source: J Cancer Res Clin Oncol. 2021 Jul 24;147(10):2993–3002. doi: 10.1007/s00432-021-03708-1 (PMC8397621; doi:10.1007/s00432-021-03708-1)
Supplement: Supplementary file 2 — Supplementary file2 (DOCX 19 KB) [file 432_2021_3708_MOESM2_ESM.docx]

**Supplementary Table 1.** Analysis of PFS and OS in subgroups

| Items | Regorafenib plus DEB-TACE | Regorafenib | PFS (months) | | | | | OS (months) | | | | |
| --- | --- | --- | --- | --- | --- | --- | --- | --- | --- | --- | --- | --- |
|  |  |  | Regorafenib plus DEB-TACE | 95%CI | Regorafenib | 95%CI | *P* value | Regorafenib plus DEB-TACE | 95%CI | Regorafenib | 95%CI | *P* value |
| Single liver metastasis | 6 | 8 | 9.5 | 7.5-11.4 | 5.5 | 3.3-7.4 | 0.002 | 18 | 13.7-22.2 | 10.7 | 9.4-13.5 | 0.005 |
| Multiple liver metastases | 28 | 34 | 7.4 | 6.6-8.1 | 3.8 | 3.6-3.9 | <0.001 | 15.2 | 13.3-17.0 | 9.5 | 9.4-9.7 | <0.001 |
| Tumor size <3 cm | 6 | 11 | 9.5 | 8.4-10.5 | 5.4 | 4.3-6.4 | 0.001 | 18 | 16.3-19.6 | 11.5 | 8.4-14.5 | 0.004 |
| Tumor size 3-5 cm | 16 | 18 | 7.5 | 7.3-7.6 | 4.1 | 3.7-4.4 | <0.001 | 15.6 | 14.6-16.5 | 9.8 | 9.5-10.0 | <0.001 |
| Tumor size >5 cm | 12 | 13 | 6.1 | 5.0-7.1 | 3.7 | 2.8-4.5 | <0.001 | 14 | 7.4-20.5 | 7.6 | 6.4-8.7 | <0.001 |

PFS, progression-free survival; OS, overall survival; DEB-TACE, drug-eluting bead transarterial chemoembolization; CI, confidence interval.
